# Autism-spectrum traits in neurotypicals predict the embodiment of manipulation knowledge about object concepts: Evidence from eyetracking

**DOI:** 10.1371/journal.pone.0268069

**Published:** 2022-07-25

**Authors:** Charles P. Davis, Inge-Marie Eigsti, Roisin Healy, Gitte H. Joergensen, Eiling Yee

**Affiliations:** 1 Department of Psychological Science, University of Connecticut, Storrs, CT, United States of America; 2 CT Institute for the Brain and Cognitive Sciences, University of Connecticut, Storrs, CT, United States of America; University of Canberra, AUSTRALIA

## Abstract

Sensorimotor-based theories of cognition predict that even subtle developmental motor differences, such as those characterizing autism spectrum disorder (ASD), impact how we represent the meaning of manipulable objects (e.g., *faucet*). Here, we test 85 neurotypical participants, who varied widely on the Adult Autism Spectrum Quotient (AQ), a measure intended to capture variability in ASD characteristics in the general adult population (participant scores were all below the clinical threshold for autism). Participants completed a visual world eyetracking task designed to assess the activation of conceptual representations of manipulable objects. Participants heard words referring to manually manipulable objects (e.g., *faucet*) while we recorded their eye movements to arrays of four objects: the named object, a related object typically manipulated similarly (e.g., *jar*), and two unrelated objects. Consistent with prior work, we observed more looks to the related object than to the unrelated ones (i.e., a manipulation-relatedness effect). This effect likely reflects the overlapping conceptual representations of objects sharing manipulation characteristics (e.g., *faucet* and *jar*), due to embodied sensorimotor properties being part of their representations. Critically, we observed—among typically developed young adults—that as AQ scores increased, manipulation-relatedness effects decreased. In contrast, in a visual control condition, in which a target object was paired with related objects of a similar shape (e.g., *snake* and *rope*), relatedness effects increased with AQ scores. The results show that AQ scores can predict variation in how object-concept representations are activated for typically developed individuals. More speculatively, they are consistent with the hypothesis that in individuals with ASD, differences in object-concept representations emerge at least in part via differences in sensorimotor experience.

## Introduction

You’re in the kitchen, washing your hands before cooking dinner, and as you turn the tap, you have a sudden craving for olives. You imagine opening the glass jar and smelling their salty aroma. Turning the faucet may have reminded you of opening a jar of olives because the two movements share action-based manipulation characteristics: we interact with (i.e., manipulate) the jar and the faucet in similar ways. Sensorimotor-based theories of cognition [1,2] suggest that our representations of object concepts are distributed over an object’s features, such that the representation of *faucet* is composed of its typical properties: *metal handle*, *smooth*, *shiny*, *is gripped*, *is turned*, *releases water*, and so on, and that those features are represented in parts of the brain actually involved in sensing, perceiving, and acting upon each feature. As a consequence of the overlapping features of *faucets* and *jars*—including the way we motorically interact with each—at least some part of your representation of *faucet* will overlap with your representation of *jar*. Importantly, this overlap is idiosyncratic—the degree to which two objects’ conceptual representations overlap depends on the degree to which an individual’s experiences with those two objects overlap. Thus, there are likely significant individual differences in representational overlap. The aim of this study was to test whether individual differences on a test of autism-spectrum traits in a non-clinical neurotypical sample are linked to differences in the motoric aspects of the conceptual representations of manipulable objects.

### Motor experience and conceptual knowledge

How an object is manipulated is one of the many features by which we represent the meaning of that object. Abundant research on semantic memory has shown that object concepts can be activated (i.e., come to mind) when a subset of the object’s features are activated (for evidence from semantic priming, see e.g., Schreuder et al. [3]; Moss et al. [4]; for evidence from the visual world paradigm, see e.g., Dahan & Tanenhaus [5]; Huettig & Altmann [6]; Yee et al. [7]). Because the representations of *faucet* and *jar* overlap in how those objects are manipulated, sensorimotor-based theories of cognition predict that thinking about the concept *faucet* will partially activate the concept *jar*, facilitating faster or more efficient access to the concept *jar*. Research to date supports this notion; Myung et al. [8] showed that people are faster to respond (in a lexical decision paradigm) to targets preceded by a word that is related in terms of how the object is manipulated (e.g., *piano–typewriter*), as compared to targets preceded by an unrelated word (e.g., *lawnmower–typewriter*). That is, shared manipulability has a priming effect. In a second experiment, Myung et al. [8] tracked participants’ eye movements to a set of four objects as they heard a word like *piano*. People were significantly more likely to look at an object that tends to be manipulated similarly (e.g., *typewriter*) as compared to a visually matched control object like *couch* or an unrelated object such as *bucket*. (Note that because objects that are manipulated similarly can also share some visual characteristics, in Myung et al. [8], these control objects were designed to address the possibility that visual similarity, rather than manipulation similarity, drove looks to the manipulation-related objects. Thus, the control and manipulation-related objects were equated with respect to visual similarity to the heard word, e.g., the visual similarity between the picture of the object referred to, piano, and the manipulation-related typewriter was equivalent to the visual similarity between piano and couch).

These “manipulation-relatedness effects” seem to be attributable to overlapping representations in areas of the brain responsible for manipulation and action. For example, although people with apraxia (a neurological condition leading to difficulty performing specific actions) do show a manipulation-relatedness effect, the effect emerges later than it does for people without apraxia, suggesting that actual motor impairments can compromise access to the manipulation-based characteristics of object concepts [9,10]. Such delayed access was not observed for visual relatedness, suggesting that the effect is specific to manipulation features [9]. Conceptually similar effects have been observed in other patient groups with deficits in action and manipulation, such as individuals with Parkinson’s disease, who show slowed processing of action-related—but critically, not abstract—words and sentences [11–13]. There is also evidence that increases in manipulation similarity are associated with increases in neural overlap in motor and action regions of the brain [14]. Specifically, when presented with pairs of words referring to objects, more similarly manipulated objects produced greater *adaptation* in left premotor cortex and intraparietal sulcus—that is, these brain regions showed a reduced response for similarly manipulated object pairs—suggesting that concepts related in manipulation similarity are represented in areas of the brain involved in guiding action.

Thus far, evidence suggests that the conceptual representations of *faucet* and *jar* are similar insofar as they tend to be manipulated similarly by *most* people, owing at least in part to similarities in the way we tend to interact with such objects. It is worth highlighting, however, that sensorimotor-based theories posit that our sensorimotor *experiences with the world* are the building blocks of our conceptual representations. Thus, according to these accounts, there should be a relationship between the *amount* and *type* of sensorimotor experience that people have with something, and *how* that thing is represented in their sensorimotor systems. There is some empirical support for this relationship—that is, the extent to which a particular sensorimotor brain system is invoked when we conceive of a given thing (e.g., a *faucet*) depends on the extent to which we have experienced that thing in (or associate it with) that modality [15–19]. Similar effects have also been observed in concept *learning*—when interacting with novel objects (which were given novel labels like “tufo”) located in the participant’s upper or lower visual field, people are faster to make responses to the learned words when the physical direction of their response (an upwards or downwards hand movement) is consistent with the physical location of that object during learning [20]. This suggests that sensorimotor experiences are encoded with words during learning. Given this evidence, we would predict that developmental sensorimotor experience will influence conceptual knowledge.

### Motor experience across the autism spectrum

Individuals on the autism spectrum, as a group, have different developmental experiences. Autism spectrum disorder (ASD) is a neurodevelopmental disorder involving atypical social skills, including difficulties with social communication, and the presence of repetitive and restrictive interests and hyper- and hypo-sensory sensitivities [21]. Importantly, ASD is seen as a continuum, with typical development at one end and the most severe symptomatology at the other. Along that continuum, there are individuals who display subclinical characteristics of ASD; while they do not display the full set of symptoms that characterize the diagnosis, they do have some mild features of ASD. This latter group is typically described as displaying the *broader autism phenotype*. Studying the broader autism phenotype is thought to lend insight into a wide range of developmental variability in otherwise typically developing (i.e., neurotypical) populations [22]. For example, family members of children with ASD show mild impairments in discourse characteristics that bear similarities to the discourse difficulties observed in ASD [23]. Other research has examined links between the broader autism phenotype and social and emotional characteristics [24], visual perceptual style [25], and the ability to ignore distractors in a visual selective attention task [26]. Variations in conceptual processing across the broader autism phenotype, however, have been less studied.

One way that conceptual processing may differ across the broader autism phenotype is in the motor aspects of conceptual knowledge. While motor deficits are not conceptualized as a defining characteristic of autism (e.g., they are not considered core symptoms of the diagnosis), autistic people (to respect the preferences of those who prefer “person-first” language—for example, “adult with ASD”—and those who prefer to highlight the centrality of ASD to their identity and thus prefer “identity-first” language, such as “autistics” or “autistic adults” [27], the current paper utilizes both approaches) display consistent motor atypicalities (noted in the very earliest descriptions; Kanner [28]) that emerge early in life and persist through development (e.g., Leonard et al. [29]). These motor deficits include—but are not limited to—impairments in movement preparation [30], movement coordination [31,32], and reaching and grasping movements [33]; an exhaustive review of motor deficits along the autism spectrum is provided elsewhere [34]. Early motor skills appear to be meaningful predictors of long-term outcomes in other, non-motor domains such as social and communication skills [35]. Such motor deficits may be attributable to atypical development of action-perception circuits [36], meaning that perceptual information is less effectively communicated to motor circuits (for further discussion, see Moseley & Pulvermüller [37]). It has also been suggested that overly precise predictive processing, in this case for motor planning, could be a source of motor deficits [38]. While some aspects of motor function, such as general gross and fine motor skills, seem to predict cognitive ability, others—namely, praxis, imitation, and coordination—uniquely predict autism-specific symptoms [39]. Importantly, motor deficits *are* observed in autistic individuals with cognitive abilities in the average range [40], providing some evidence that motor skills are associated with some of the hallmark differences that characterize the autism spectrum. Indeed, individuals with sub-clinical autism characteristics show differences, measured via electrophysiological brain responses, in how they perceive motoric actions upon objects [41], suggesting that the motoric differences that characterize autism may not be limited to full clinical autism, but rather, are present across the spectrum.

Developmental differences in motor experience are increasingly recognized as a hallmark of the autism spectrum [36,37,42,43]. For example, affordance perception refers to the understanding of possibilities for action in the environment, such as whether an object can be grasped or sat on [44]. Impaired affordance perception appears to be one consequence of these motor atypicalities in ASD. Errors in recognizing reachability (whether an object can be reached in its current position), graspability (whether an object can be grasped with a particular hand position), and aperture (whether one’s hand can fit through a hole of a given size) were found to be more frequent among autistic adolescents and adults who have cognitive abilities in the average range, as compared to typically developing age- and IQ-matched control participants [45]. Error rates were correlated with the severity of social and communicative difficulties, suggesting a specific link between affordance abilities and autism spectrum symptoms. This suggests that the autism spectrum is characterized by atypicalities in the integration of perceptual and motor information, and specifically, atypicalities in the perception of information in the environment as it relates to one’s body. Differences in motor ability, then, impact how the environment can be experienced via manipulation and action, and it is these differences in experience that we contend underpin possible differences in conceptual knowledge along the autism spectrum. As discussed in this section, these differences appear even in adults with intact cognitive abilities.

### Motor experience and conceptual knowledge in ASD

Recent theories of motor deficits on the autism spectrum suggest that such deficits may have implications for conceptual processing (e.g., Eigsti [42]; Moseley & Pulvermüller [37]). Given the important role of sensorimotor experience for early word learning (e.g., Yu & Smith [46]), early motor differences could impact the influence of sensorimotor properties in conceptual representations; for example, concepts may be less bound to the sensorimotor conditions that held at concept acquisition [42]. There is evidence supporting this proposal from a study [47] showing that ASD modulates the impact of motor action on the meanings that people associate with novel images, such as Japanese kanji characters. In this two-phase experiment, individuals with and without ASD—all with cognitive abilities in the average range—were examined. Participants, who had no prior experience with Japanese, were first exposed to Japanese kanji characters (e.g., 英) while posing in either an approach (flexion) or avoidance (tension) position. In prior work, it was found that holding these postures elicited more positive (approach) versus less positive (avoidance) attitudes towards previously neutral stimuli [48]. In a second phase, for each kanji character, participants were asked to give their best guess as to which of two images (one positive, one neutral) was most likely to depict the kanji’s meaning. While participants without ASD matched kanji characters that had been encoded while posed in an approach position with more positively valenced pictures, this was *not* the case for participants with ASD, for whom there was no relationship between their posture when encoding a character and the valence of the picture to which they matched that character [47]. These results suggest that while the motor states involved in encoding a novel stimulus persist when a typically developing person later re-encounters that stimulus (see also [20]), this may not be the case for people with ASD.

In summary, there is mounting evidence that motor atypicalities are a central feature of the autism spectrum, and that such atypicalities have implications for conceptual processing. Some work has also investigated the role that autism traits—and presumably, developmental motor atypicalities—may have in processing action concepts (e.g., *kick*). An imaging study of adults with ASD found that delays in the semantic processing of action words were tied to reduced activation of motor cortex, and also tied to symptom severity [49]. Adults high in autism-spectrum characteristics also showed atypical motor-related brain activity when viewing pictures of manipulable objects, perhaps reflecting deficits in processing motor affordances [50].

However, to our knowledge, the relationship between autism-spectrum characteristics in neurotypical individuals and the manipulation characteristics of object concepts has never been investigated. This is the focus of the current work. We reasoned that if motoric affordance information of objects is indeed less robustly encoded by those on the autism spectrum [45,50] and their motor systems are less active when processing action-based semantic information [49], then even among neurotypical individuals, the representation and processing of object concepts (e.g., *jar*) should be less reliant on experience-based manipulation information (what it *feels* like to open a jar) as autism-spectrum traits increase.

### The present study

In this experiment, we investigated whether a measure of autism-spectrum traits predicts the degree to which manipulation-based conceptual knowledge is activated in conceptual processing. Motor differences seem to emerge across the autism spectrum (e.g., [45,51]) and persist into adulthood (e.g., [45]), suggesting that differences in the activation of manipulation-based conceptual knowledge should be continuous across the autism spectrum, and should be present even in a non-clinical, young adult sample.

We used the visual world paradigm (e.g., Allopenna et al. [52]; Tanenhaus et al. [53]) to probe on-line conceptual processing (e.g., Yee & Sedivy [54]) in young adults. The visual world paradigm has been shown to be a reliable measure for testing individual differences [55,56], and thus may be a useful paradigm for studying subtle differences in semantic access in different populations (e.g., [9,10,57,58]). We employed a commonly used variant of the visual world paradigm in which participants are presented with an array of objects and their eye movements are tracked as they listen to a spoken “target” word. Typically, participants are asked to perform an action on the object referred to by the target word. Although subjects’ eye movements ultimately settle on the target object (e.g., *faucet*) in most trials, pre-decision eye movements to non-target distractor objects (e.g., *jar*) are taken to reflect partial activation of those objects resulting from hearing a word referring to the target object (e.g., *faucet*).

We preregistered the prediction (https://osf.io/7fzsu/) that, if subclinical autism-spectrum traits are indeed related to subtle differences in motor experience, then these traits should be associated with reduced manipulation-relatedness effects in a visual world paradigm. That is, hearing the word *jar*, people with higher autism-spectrum traits should look less to *faucet* as compared to people with lower levels of ASD traits. We anticipated that the reduced relatedness effect would be specific to manipulation characteristics, such that there would be no reduction in responses to objects that were related in shape. The distinction between our predictions for manipulation vs. shape relatedness is important—a reduction in manipulation-relatedness effects without an appropriate control could simply reflect abnormal semantic processing more broadly (e.g., Kamio et al. [59]; but see Hala et al. [60]), rather than a difference more plausibly related to motor experience.

## Methods

### Participants

Participants were 101 undergraduate students at the University of Connecticut (77 women, 24 men; *M*_age_ = 18.67 years, range = 17–21) who provided written informed consent prior to testing and were compensated with course credit. Here, we deviated from our preregistration, where we indicated that we would run 40 participants based on a power analysis for a 2 × 2 between-within factorial design. After collecting 40 participants, we examined the distribution of AQ scores (we did not examine eyetracking results). As there was insufficient variability to split participants into high- and low-AQ groups as a between-subjects variable, we decided to instead use AQ scores as continuous predictor, and increased the sample size to maximize AQ score range and variability (i.e., to obtain a normally distributed range of AQ scores across the sub-clinical range; data collection concluded when this distribution was obtained). No participants self-reported a diagnosis of ASD, but we did not explicitly ask about the diagnosis. All participants reported having normal or corrected-to-normal vision, and no hearing deficits. The experimental procedures were approved by the University of Connecticut Institutional Review Board.

### Materials

#### Adult autism-spectrum quotient

Participants completed the Adult Autism-Spectrum Quotient (AQ) [61] as a measure of autism-spectrum traits. The AQ is a 50-item measure of autism traits, where the total score (maximum = 50) is taken to reflect a continuous measure of traits along the autism spectrum; this measure has been used in over 500 publications to date as a means of capturing subclinical ASD characteristics in neurotypical (i.e., not autistic) populations. Higher scores indicate a greater degree of autism-spectrum traits. While a score ≥ 32 is taken to reflect clinically significant autism traits, variation across the entire scale reflects meaningful differences in autism-spectrum characteristics. The average AQ score in our sample was 18.10 (range = 7–31, *SD* = 5.57).

#### Visual world task

**Fig 1:**
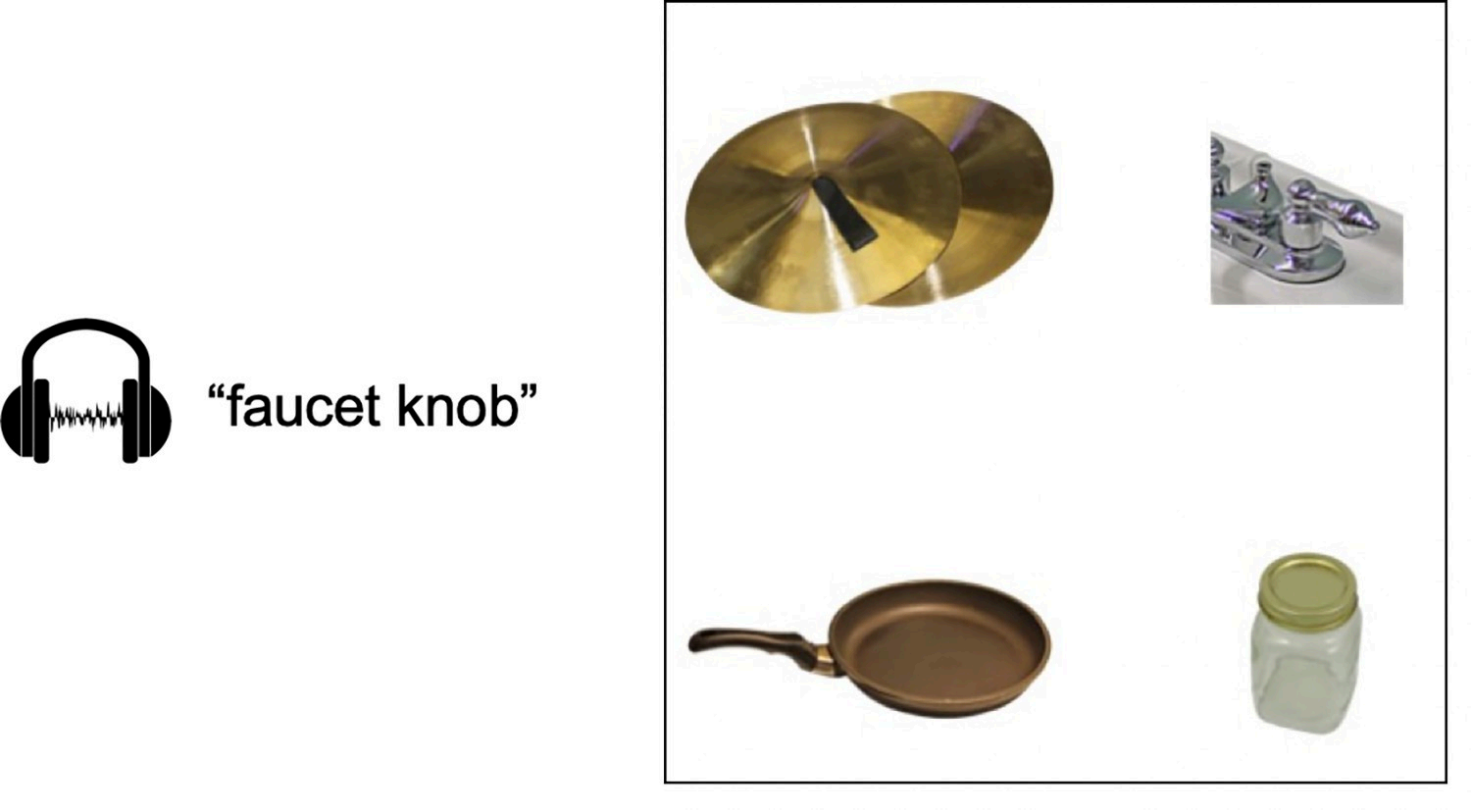
Example of the visual world setup. Object positions were balanced such that each object type was equally likely to appear in each corner of the display. Actual images were derived primarily from the Bank of Standardized Stimuli (BOSS [62]). Images in the visual world are republished from Brodeur et al. [62] under a CC BY license, with permission from Matthieu Brodeur, original copyright (2009, 2010).

Each trial included four objects (Fig 1). After a 1-second preview of the array, the name of the target object was presented auditorily. Upon hearing the target (e.g., “*faucet knob*”), participants clicked on the object to which the word referred as quickly and accurately as possible. After the participant clicked on an object, the trial ended. Each trial was followed by a fixation cross displayed for 500 ms.

There were three trial types in this experiment: 24 *manipulation-*related trials (where one distractor object shared manipulation characteristics with the target; e.g., *faucet knob*–*jar*, while the other two were unrelated), 24 *shape*-related trials (where one distractor object shared shape characteristics with the target, e.g., *rope*–*snake*, while the other two were unrelated), and 48 filler trials (where none of the three distractor objects were related to the target). Trial type was manipulated within-subjects, and no objects were repeated (e.g., the unrelated objects were different between manipulation- and shape-related trials; all stimuli are shown in the Appendix.) Object positions were balanced such that each object type was equally likely to appear in each corner of the display, and stimuli were presented in a fixed random order. Stimuli were mostly gathered from previous similar studies—specifically, manipulation-relatedness trials were generated based on the stimuli of Myung et al. [8,9] and shape-relatedness trials were based on Dahan and Tanenhaus [5].

To ensure that all manipulation-related items were highly similar on *manipulation* but not *shape* relatedness, and that all shape-related items were rated as highly similar in shape but not manipulation relatedness, norming was conducted with a sample of 67 University of Connecticut undergraduates who did not participate in the eyetracking study (*N* = 34 for manipulation-relatedness and *N* = 33 for shape-relatedness). Norming participants were compensated with course credit. For each pair of words, participants were asked to respond using a 7-point Likert-type scale, as follows: for manipulation-relatedness, “How similar are the movements you make when you interact with these objects?”; for shape-relatedness, “How similar are the shapes of these objects?”. The 24 manipulation-related pairs that were included in the eyetracking study had a mean manipulation-relatedness rating of 5.07 (*SD* = 0.533; range = 4.24–6.09) and a mean shape-relatedness rating of 1.76 (*SD* = 0.889; range = 0.39–3.21), a difference that was statistically reliable (Welch’s *t* = 14.29, *p* < .001). The 24 shape-related pairs had a mean shape-relatedness rating of 4.40 (*SD* = 0.682; range = 2.88–5.36) and a mean manipulation-relatedness rating of 2.50 (*SD* = 0.702; range = 1.29–3.88), a difference that was also statistically reliable (*t* = -11.56, *p* < .001).

### Apparatus

An Eyelink 1000 Plus eyetracker (SR Research, Ltd., Ontario, Canada) was used to monitor participants’ eye movements. The eyetracker sampled at 500 Hz from the right eye (because eye movements are yoked, and most people are right-eye dominant, it is standard to track only the right eye in visual world paradigm studies). Stimuli were presented using Experiment Builder software (SR Research, Ltd., Ontario, Canada), which also collected response data. Participants were comfortably seated in front of a 24” LED monitor with their eyes approximately 60 cm from the display. Images varied slightly in size, but on average, each imaged subtended roughly 5–6° of visual angle. The eyetracker is accurate to less than 1° of visual angle.

### Procedure

After completing written informed consent, participants were asked to follow a standard 5-step calibration procedure. To take part in the experiment, a participant’s average calibration error had to be less than 1.5°, with their worst point error less than 2° (this corresponds to a “good” or “fair” calibration grade in Eyelink software). Participants then completed four practice trials (none of which included shape- or manipulation-related objects) to ensure familiarity with the task procedures and timing. Following each trial, participants were instructed to fixate on a centered black dot, which was used to correct calibration drift. The mouse cursor reset to the center of the screen at the beginning of each trial in order to reorient participants to the center of the screen and reduce variability in RTs that would have resulted from inconsistent starting points. After the visual world experiment, they completed a computerized version of the AQ, which was simply introduced as a short questionnaire.

### Data analysis

Data were analyzed in R 4.0.5 [63]. Eye-movement data were preprocessed in part using a selection of functions from the *gazeR* package [64], and processed in 10-ms bins from word onset until 1200 ms post-stimulus, at which point looks to the target were no longer increasing. The *ggplot2* package [65] was used for data visualization. The raw data, analysis scripts, and stimuli are available on Open Science Framework (https://osf.io/7fzsu/).

**Fig 2:**
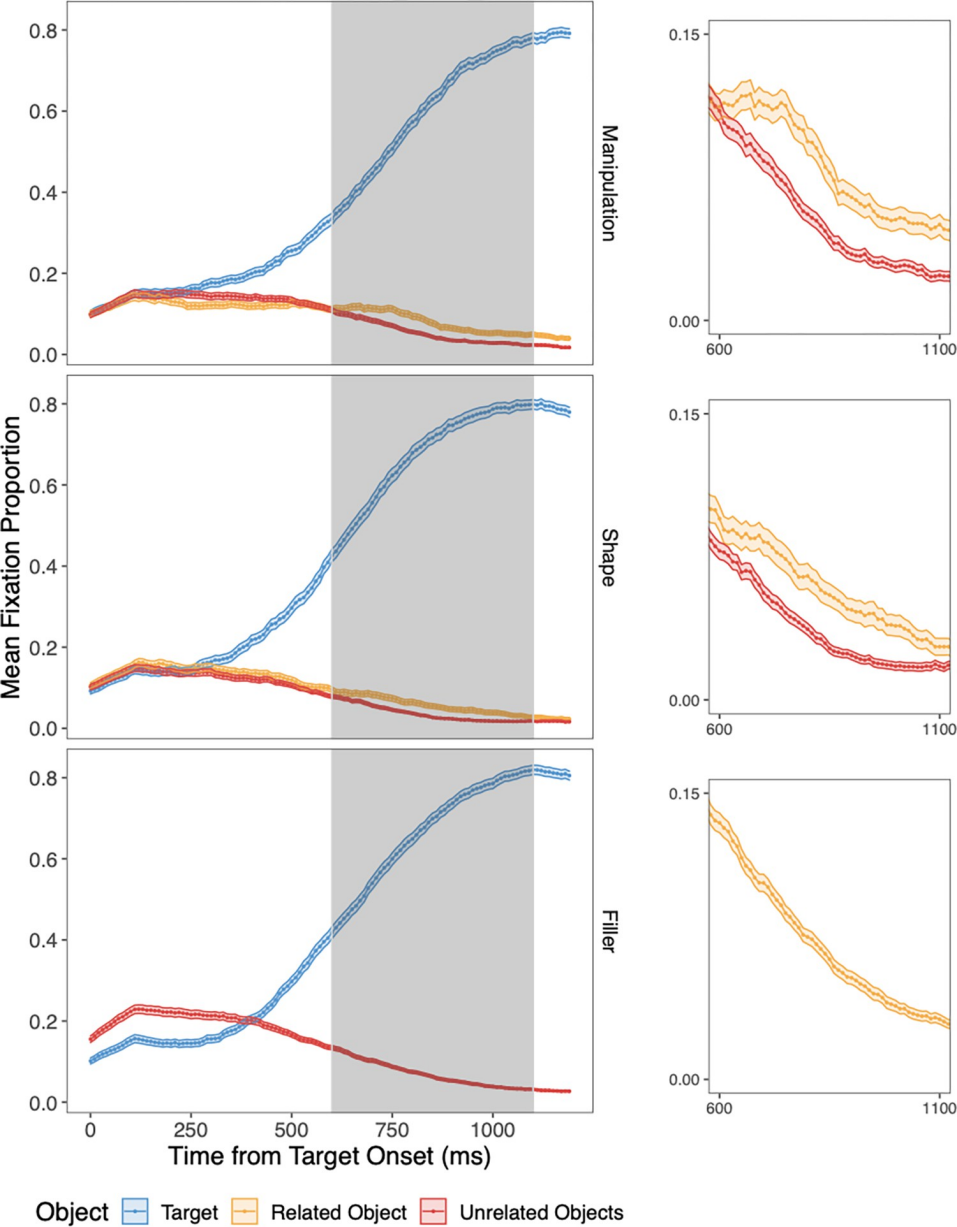
Mean proportion of fixations over time from word onset (in 10-ms bins, averaged across participants) for manipulation trials (top), shape trials (middle), and filler trials (bottom). Blue lines indicate looks to the target, yellow lines indicate looks to the related item (for manipulation and shape trials), and red lines indicate looks to the average of two unrelated items. Bands around each line represent the standard error at each 10-ms bin. The gray area denotes the window within which relatedness effects were analyzed, and the inset graphs show a zoomed-in view of the relatedness effect during that time window, which—as expected—is evident for both shape and manipulation trials, but not fillers.

We evaluated our hypotheses using by-subject linear regression models. All analyses were conducted by subject because our main interest was in the relationship between a subject-level individual differences measure (i.e., the AQ) and the size of the “relatedness effect.” To operationalize relatedness effects, we first visually inspected the data to determine when they began to emerge for manipulation trials and for shape trials. This was around 600 ms for both (see Fig 2). After 1100 ms, looks to the target did not increase by more than 1% in any subsequent 100-ms bin, so the window of analysis ended at 1100 ms. Within this window, we first took the average proportion of fixations for each object in each trial. We then averaged this proportion across all trials in a given condition for each participant. Next, for each participant, we measured the relatedness effect (defined as the average proportion of fixations to the related object minus the averaged proportion of looks to the two unrelated distractor objects) in the window from 600 to 1100 ms. (Note that similar—and also statistically reliable—results to those presented here are obtained when using a more formal approach where the competitor effect window is defined as starting in the time bin at which fixations to the competitor and distractors reliably diverge [710 ms] and ending when they converge [1100 ms].) This period is similar to the timecourse of the relatedness effects observed in Myung et al. [8], who used a similar procedure (e.g., in Fig 2 of Myung et al. [8] the manipulation-relatedness effect appears at about 500–800 ms). Responses during this window were used to test the effects of our orthogonal predictor—AQ scores—on manipulation- and shape-relatedness effects.

Model construction proceeded in three steps. (1) We first constructed a main effects model including the main effects for Trial Type (shape or manipulation) and AQ Score. (2) Then, our critical hypothesis—that there is an interaction between Trial Type and AQ Score, such that higher AQ scores are associated with smaller manipulation (but not shape) relatedness effects—was evaluated by adding the Trial Type × AQ Score interaction. (3) We also constructed an exploratory model exploring the hypothesis that less efficient motor skills might contribute to differences in manipulation-relatedness effects. We used button-press RT as a proxy for motor efficiency—longer button-press RTs should reflect reduced motor efficiency—and added a Trial Type × Button-Press RT interaction to the previous model (only RTs from filler trials were included because RTs from trials with related items may be influenced by looks to those items, though RTs for all trial types are available in the Online Supplemental Material). The statistical significance of each model was evaluated using a model comparison approach, and *p*-values less than .05 were considered statistically significant.

## Results

Thirteen participants were excluded due to poor calibration (typically because of dark makeup around the eyes), two were excluded due to having button-press response times (RTs) greater than 2.5 *SD* from the mean (these participants routinely showed RTs > 10 s, reflecting a lack of attention or other confounding factors), and another was excluded due to having non-fixation times (i.e., proportion of time not looking at any of the four objects) greater than 2.5 *SD* from the mean, leaving *N* = 85. The pattern of results is unchanged if these participants are included in analyses, but because of concerns that they may not have engaged with the task as directed, they are omitted from reported analyses.

As is typical in visual world studies (e.g., Yee et al. [7]), fixations were only included if the eyes remained on an object for at least 100 ms, and if the fixation began before a response was made (i.e., before the target was clicked on). We removed trials with RTs under 150 ms and over 5000 ms (1.9% of all trials), as these reflected either insufficient time to hear the word or a lack of attention, respectively. We also removed trials where the correct object was not selected (0.7% of all trials), as these likely reflected inattention to the spoken stimulus. The overall mean RT in the cleaned data was 1666 ms (*SD* = 260 ms), and the RT breakdown by condition was as follows: 1762 ms (*SD* = 272 ms) for manipulation trials, 1566 ms (*SD* = 233 ms) for shape trials, and 1669 ms (*SD* = 237 ms) for filler trials. To visualize the timecourse of eye movements, Fig 2 shows, for each trial type (manipulation, shape, and filler), the mean proportion of trials over time containing a fixation to each object. The relatedness effect time window is highlighted in gray.

Our primary analysis concerned the impact of autism-spectrum characteristics (as measured by AQ) on relatedness effects—in particular, whether an increase in AQ scores would be associated with a decrease in manipulation-relatedness effects (relative to shape-relatedness effects). Thus, we were concerned with the relation between the manipulation- and shape-relatedness effects and AQ score, rather than the absolute magnitudes of the relatedness effects. In the main effects model, there were no main effects of Trial Type (i.e., manipulation and shape effects did not differ from each other; *t* = -.92, *p* = .359), or of AQ Score (*t* = -.22, *p* = .829).

**Fig 3:**
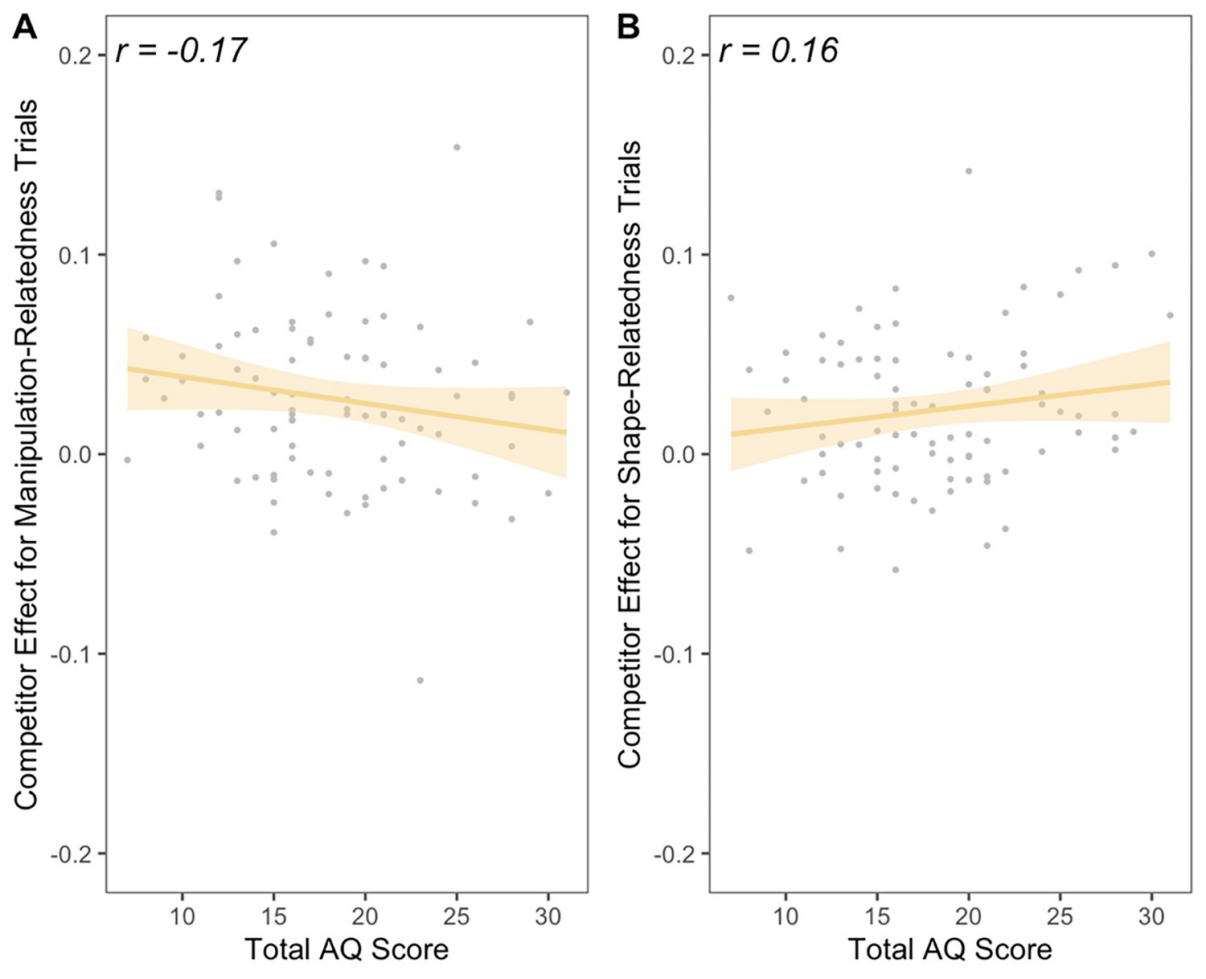
Correlation between total AQ score and the relatedness effect (proportion fixations to related item–proportion fixations to unrelated items) for (A) trials with a manipulation-related object (e.g., *faucet–jar*) and (B) trials with a shape-related object (e.g., *rope–snake*). The relatedness effect was calculated as the average of fixation proportions in the critical 600- to 1100-ms time window (see Fig 2). Each dot (in each panel) represents one subject. Pearson’s *r* correlations are superimposed on each panel.

In the interaction model, we observed a significant Trial Type × AQ Score interaction (*t* = 2.16, *p* = .032), as predicted. The model comparison showed that the interaction model was significantly more predictive than the main effects model (*F*(1, 166) = 4.67, *p* = .032). In line with our predictions, the manipulation-relatedness effect *decreased* as AQ scores increased, suggesting that individuals higher in ASD traits showed reduced relatedness effects during manipulation trials (Fig 3). Also in line with our predictions, the shape-relatedness effect did not decrease with increasing AQ scores (in fact, the shape-relatedness effect increased as AQ scores increased). Full model results are detailed in Table 1.

**Table 1:** Model Results Predicting Relatedness Effects from AQ and Competitor Type.

	Estimate	SE	t-value	p-value
Model 1				
Trial Type	-0.006	0.006	-0.92	.359
AQ Score	0.0001	0.001	-0.22	.829
Model 2				
Trial Type	-0.050	0.021	-2.34	.021
AQ Score	-0.001	0.001	-1.68	.094
Trial Type × AQ Score	0.002	0.001	2.16	.032
Exploratory Model				
Trial Type	-0.147	0.046	-3.20	.002
AQ Score	-0.001	0.0008	-1.65	.101
Button-Press RT	-0.00001	0.00002	-0.58	.566
Trial Type × AQ Score	0.002	0.001	1.97	.050
Trial Type × Button-Press RT	0.00006	0.00003	2.38	.019

The exploratory model using button-press RTs as a proxy for motor efficiency showed a significant Trial Type × Button-Press RT interaction (*t* = 2.38, *p* = .019), such that as RT increased, manipulation-trial relatedness effects decreased slightly and shape-trial relatedness effects increased considerably. The model was significantly more predictive than was the critical interaction model (*F*(1,164) = 5.65, p = .019), suggesting that motor efficiency may indeed have contributed to relatedness effects. Specifically, this suggests that people who are slower to make button-press responses, a rough proxy of motor efficiency, might rely less on manual information than they do on visual information in representing concept meanings. Finally, even after adding Button-Press RT, the Trial Type × AQ interaction remained statistically significant (see Table 1 for detailed model results; note that while the reported exploratory models use raw RTs, the same results were obtained using logged RTs).

## Discussion

Higher scores on the Adult Autism Spectrum Quotient (AQ) in neurotypicals were associated with reduced manipulation-relatedness effects—that is, upon hearing “faucet,” the activation of *jar* became increasingly unlikely as autism-spectrum traits increased. Faced with the task of turning on a faucet, individuals with higher AQ scores may show decreases in the likelihood that they think about opening a jar. On the other hand, as AQ scores increase, seeing a pile of rope on the garage floor is no less likely (and in fact, is somewhat *more* likely) to activate a visually similar concept such as *snake*. In the following, we consider the implications of this finding for how conceptual knowledge is represented along the broader autism phenotype, as well as the general implications for theories of concept representation.

By showing that even among non-autistic individuals, scoring higher on a test of autism traits is associated with subtle differences in the activation of manipulation knowledge, our results suggest that the broader autism phenotype as observed in the general population is associated with differences in the representation of conceptual knowledge. Studies suggest that individuals with ASD represent conceptual knowledge differently, showing differences in semantic priming [59] and in the neural processing of abstract emotion words [66] and action-related words [49]. Further, these differences may be attributable to delays and deficits in the development of motor skills on the autism spectrum (see Eigsti [42]; Moseley & Pulvermüller [37]), which are increasingly being recognized as a central feature of the diagnosis [36,43].

The present study extends findings of atypical object-concept representations from the full clinical ASD presentation to the broader autism phenotype, adding to growing evidence that many traits that characterize ASD are also observable in typically developing populations as the broader autism phenotype (see Landry & Chouinard [22]). Notably, the present findings—i.e., that the range of experiences associated with autism-spectrum traits predicts differences in the motoric bases of object-concept representations—do not necessarily indicate a *deficit* in processing; rather, they could be conceptualized as a greater resistance to competition effects, greater independence of semantic representations, or simply *differences* in semantic representations (i.e., differences in the way in which the manipulation properties of objects are activated).

In fact, the finding that shape-relatedness effects become larger as autism-spectrum traits increase is consistent with the presence of *conceptual compensation* (or adaptation), such that if information in one modality is less informative (or even absent), information from other modalities (e.g., vision) becomes more prominent in representations (see Yee et al. [67] for discussion). This kind of compensation could help explain why, for example, individuals who are born with severely shortened or absent upper limbs can recognize, remember, and anticipate actions as accurately as typically developed participants [68]. For these individuals, action knowledge may be supported by the experiences that they do have with actions, which would include visual experiences. This finding is also consistent with the suggestion that visual information is highly salient during language processing in people with ASD [69]. That said, although our exploratory analysis using button-press RT (from filler trials) as a proxy for motor efficiency is consistent with our interpretation—in that as RTs go up, the manipulation-relatedness effect goes down and the shape-relatedness effect goes up—it is not without caveats. In particular, one might predict that if motor efficiency underlies the relationship between autism-spectrum traits and our measure of conceptual knowledge (i.e., relatedness effects), then including a measure of motor efficiency in the model might *weaken* the relationship between autism spectrum traits and relatedness effects by absorbing some of the variance that had been attributed to AQ scores. However, the relationship between autism-spectrum traits and relatedness effects was only very slightly (non-significantly) weakened when button-press RT was included in the model. It is possible that this is because autism spectrum traits are associated with motor inefficiencies that are not captured by button-press RT, an admittedly very rough proxy of motor efficiency.

The present findings also lend further support to experience-based theories of semantic memory—namely, sensorimotor-based models—suggesting that individual differences in developmental experience (in the present study, those that characterize the broader autism phenotype) predict differences in the way conceptual knowledge is represented and activated (for discussion, see [70]). This adds to a growing literature showing that the way objects are typically experienced (e.g., [17,19]) and differences in expertise with objects (e.g., [71,72]) drive how concepts are represented, and that when those objects afford manipulation, they activate action-related brain areas (e.g., [73]). For example, a barista might have a robust representation of *faucet* that is more likely to activate manipulation-related knowledge relevant to coffee, like turning on a steam wand or opening a freshly roasted jar of coffee beans. There is considerable evidence that adverse later-life events (e.g., brain injury, neuropsychological conditions) can impact conceptual representations in the affected modality (e.g., motor function in apraxia and Parkinson’s disease [9–13]). Differences in developmental experience also affect concept representation—right-handers, to a greater degree than left-handers, seem to represent the meaning of manipulable objects in the contralateral premotor cortex, a difference not observed for non-manipulable objects [74,75]. But sensorimotor-based theories of cognition predict that even more subtle differences in experience should affect the way concept representations develop. Here, we have provided evidence that this is indeed the case.

### Limitations

We have shown that even in a non-referred, non-clinical population, participants with greater levels of autism-spectrum traits—which exist on a continuum even in neurotypical individuals—appear to show reduced grounding in motor experience of object-concept representations. Our conclusions about the role of autism traits (and, as we assume, subtle motor atypicalities) are limited by two factors. First, no participants reported a history of a clinical ASD diagnosis, and overall, participants reported relatively low levels of ASD traits on the AQ (mean score of 18.1, range of 7–31, where scores reflect more traits on this questionnaire and 32 is the consensus threshold for ASD). It remains to be seen whether these differences in object-concept representations are indeed a characteristic of ASD; data from a definitively diagnosed sample will be required to fully address this possibility. We speculate that the fact that our participants had relatively low levels of ASD traits may be part of why the observed effects were quite small, and that a clinical sample of individuals with ASD would yield a larger effect, clarifying the link between autism traits and activation of object-concept representations.

A second important limitation concerns the lack of a direct assessment of motor function, making it difficult to draw a direct link between the developmental motor issues that might characterize the broader autism phenotype and differences in the motoric bases of object-concept representations. We suspect that any motor function deficits present in our participant population would be too subtle to be detected using available standardized assessments for adults, which are largely aimed at detecting major motor skills impairments. Our exploratory analysis using button-press RTs as a proxy for motor efficiency is suggestive, but a carefully designed task that is sensitive to subtle variation in motor skills is needed. Indeed, we would expect that using a direct measure of motor function as a predictor of differences in conceptual knowledge would yield larger differences—as noted above, effects were quite small. Such a measure, in combination with the kind of semantic processing task used here, would enable explicit testing of the hypothesis that developmental motor atypicalities underpin differences in conceptual knowledge along the autism spectrum. Although further work is needed, it is noteworthy that sensitivity to similarity in how objects are manipulated decreased as a function of autism-spectrum traits, whereas there was no such decrease (and in fact, there was an increase) in sensitivity to similarity in shape. Together, these findings suggest that the source of the difference is relatively specific.

A final caveat stems from the fact that we neither controlled for participants’ handedness nor for the orientation of objects’ handles in the visual displays. Given that grasp information about an object is more readily available when handedness and the orientation of an object’s handle match [76], and that autism-spectrum disorder has been associated with mixed handedness (for review, see [77]), an imbalance of handle orientation among our items could have impacted our findings: Specifically, if most objects had handles oriented to the right, manipulation information could have less salient for participants with more autism-spectrum traits. To explore this possibility, we inspected the manipulation-related items and found that for 7 of the 24, handle orientation to the right could have decreased manipulation salience for non-right-handed participants. An analysis of the data with these 7 items excluded showed the same pattern of results as in the full dataset, suggesting that handle orientation did not interact with our effects.

## Conclusions

In sum, the present findings converge with two bodies of literature, one showing that conceptual embodiment is atypical along the autism spectrum (for reviews, see [37,42]), and the other demonstrating the critical role of sensorimotor *experience* in building conceptual representations (for demonstrations, see e.g., [17,19]). The results are consistent with the hypothesis that experiences characterizing the broader autism phenotype engender differences in object-concept representations. That is, in a population exhibiting typical variability on a measure of autism-spectrum traits, individual differences predict the likelihood that one will think of a jar of olives when turning on the faucet. These findings shed light on experiential factors that influence the richly interconnected semantic networks underpinning object and action representations.

## Appendix

**Table A1:** Manipulation and Shape Trial Stimuli.

Stimulus #	Target (Heard)	Related	Unrelated	Unrelated
*Manipulation*				
MAN01	axe	baseball_bat	watermelon	whiteout
MAN02	whistle	balloon	plate	zipper
MAN03	lollipop	pacifier	pencil	ironing_board
MAN04	stopwatch	lighter	notebook	jug
MAN05	violin_bow	iron	binoculars	barrel
MAN06	bracelet	glove	tube	watering_can
MAN07	baby_carriage	lawn_mower	car	apron
MAN08	camera	perfume	plunger	pitcher
MAN09	butcher_knife	hammer	flower	dice
MAN10	clothes_pin	nail_clippers	medallion	hand_grip
MAN11	eye_drops	tweezers	umbrella	muffin_tin
MAN12	doorbell	vending_machine	comb	spindle
MAN13	sandpaper	sponge	shell	remote
MAN14	spear	football	salt_shaker	leaf
MAN15	key	screwdriver	envelope	set_square
MAN16	shoelace	corset	shovel	eye_mask
MAN17	paint_roller	pizza_cutter	toothbrush	sunglasses
MAN18	toilet_handle	toaster	magnet	easel
MAN19	butterfly_net	flag	pill	razor
MAN20	typewriter	piano	shirt	ruler
MAN21	buzzer_button	handsoap_pump	cigar	bow
MAN22	faucet_knob	jar_lid	pan	cymbals
MAN23	whip	maracas	scissors	candlestick
MAN24	twist_tie	can_opener	wrench	birdie
*Shape*				
VIS01	acorn	dreidel	tissue_box	microphone
VIS02	frisbee	CD	mailbox	mallet
VIS03	cane	fishing_hook	coins	tongs
VIS04	clipboard	playing_card	helmet	airhorn
VIS05	magnifying_glass	pingpong_paddle	suitcase	fedora
VIS06	martini_glass	funnel	doorknob	recorder
VIS07	hockey_puck	biscuit	table	cotton_swab
VIS08	ring	donut	horseshoe	cork
VIS09	hot_air_balloon	lightbulb	spray_bottle	battery
VIS10	wheel	lemon	basket	paper_towels
VIS11	carrot	torch	spatula	teddy_bear
VIS12	hourglass	bowtie	broom	anchor
VIS13	purse	padlock	lego	bottle_cap
VIS14	mushroom	lamp	teabag	music_stand
VIS15	cherry	bomb	hairdryer	towel
VIS16	bottle	bowling_pin	lipstick	cheese_grater
VIS17	golfball	orange	clock	handsaw
VIS18	traffic_cone	witch_hat	pliers	tobacco_pipe
VIS19	rope	snake	tie	sardine_can
VIS20	school_bus	loaf_of_bread	hedge_trimmers	paintbrush
VIS21	flower_pot	top_hat	paint_pallet	fortune_cookie
VIS22	turtle	igloo	hanger	drum
VIS23	train	caterpillar	toilet_brush	tape_roll
VIS24	winter_hat	bell	folder	flash_drive

*Note*. All picture materials can be found in the Supplemental Material, available on the Open Science Framework (https://osf.io/7fzsu/). The stimulus code provided in the first column here corresponds to the stimulus code for each picture stimulus filename online. In the picture filenames, T = target, C = related competitor, and D1 and D2 = unrelated distractor items.
